# A Classification Method for Select Defects in Power Transformers Based on the Acoustic Signals

**DOI:** 10.3390/s19235212

**Published:** 2019-11-28

**Authors:** Michał Kunicki, Daria Wotzka

**Affiliations:** 1 Institute of Electrical Power Engineering and Renewable Energy, Opole University of Technology, 45-758 Opole, Poland; 2 Faculty of Electrical Engineering Automatic Control and Informatics, Institute of Automatic Control, Opole University of Technology, 45-758 Opole, Poland; d.wotzka@po.edu.pl

**Keywords:** partial discharges, condition monitoring, acoustic emission, power transformer

## Abstract

Effective, accurate and adequately early detection of any potential defects in power transformers is still a challenging issue. As the acoustic method is known as one of the noninvasive and nondestructive testing methods, this paper proposes a new approach of the classification method for defect identification in power transformers based on the acoustic measurements. Typical application of acoustic emission (AE) method is the detection of partial discharges (PD); however, during PD measurements other defects may also be identified in the transformer. In this research, a database of various signal sources recorded during acoustic PD measurements in real-life power transformers over several years was gathered. Furthermore, all of the signals are divided into two groups (PD/other) and in the second step into eight classes of various defects. Based on these, selected classification models including machine learning algorithms were applied to training and validation. Energy patterns based on the discrete wavelet transform (DWT) were used as model inputs. As a result, the presented method allows one to identify with high accuracy, not only the selected kind of PD (1st step), but other kinds of faults or anomalies within the transformer being tested (2nd step). The proposed two-step classification method may be applied as a supplementary part of a technical condition assessment system or decision support system for management of power transformers.

## 1. Introduction

Adequate, accurate and early defect detection is one of the key issues related to fleet maintenance of power transformers [1,2]. Several different diagnostic methods are commonly used to assess the technical condition of transformers [3,4,5,6,7,8,9]. In particular, the most important from the point of view of operational safety is the assessment of the technical condition of the insulation system. One of the biggest threats to high voltage insulation systems is the partial discharge (PD). Currently, many methods of measuring and assessing PD are known, but three of them are mainly used in industrial practice: the electrical method, the ultra-high frequency (UHF) method and the acoustic emission (AE) method [10,11,12,13,14]. Each of these methods is characterized by some strengths and weaknesses: for example, only the electrical method can be calibrated (delivers direct information about apparent charge amplitudes), but it is also very susceptible to interference from the grid or the substation surrounding apparatus. The UHF method is quite resistant to disturbances but requires placing the sensor inside the tank (directly in the oil) and cannot be calibrated so far (despite that intensive research are conducted in that matter). Regarding the AE method, it is the only fully noninvasive PD measuring method that may be applied during normal exploitation conditions [15,16]. It also should be emphasized that any disconnection of the transformer is associated with the necessity to periodically change the configuration of the power grid or interrupt the supply of power to consumers, so the mentioned property of the AE method is significant for the diagnostic process. Nevertheless, the AE method has also a few disadvantages that should be highlighted: it is much less sensitive than the UHF and electrical methods and it is prone to external noise [17,18,19]. In addition, usually, not all PD defects are detectable using the AE method. For example, acoustic signals emitted by PD located around the internal parts of the windings are difficult to register by the commonly applied transducers, due to the high attenuation of the acoustic wave in transformer oil [20]. Only about 1% of the AE signal energy reaching the tank surface is transmitted to the sensor; 99% is reflected due to the impedance mismatch between the oil and steel. Oil-immersed sensors (e.g., hydrophones) are sometimes used to solve this issue, but the possibility of placing them inside the transformer tank is still not optimal in terms of the potential efficiency of the method [21,22].

PD measurements using the AE method under on-site, normal operating conditions are always affected by a number of external and internal disturbances that are expected within the tested unit. To ensure the correct interpretation of the recorded signals, it is necessary to distinguish the signals generated by the PD from the other signals. Many contemporary research papers concern the issue of denoising AE signals. Different algorithms have been proposed, usually based on artificial neural networks, fuzzy logic or wavelet decomposition [23,24,25,26,27,28]. Unfortunately most of the researchers used AE signals emitted by artificial PD sources in a laboratory environment, and usually, no real-life scenario was presented to verify the proposed methods. Identifying (or classifying) the source of PD using the AE method is another issue readily discussed in modern research [18,26,27,29,30,31]. Additionally, in this case, it is usually limited to several selected artificial PD defects, generated under laboratory conditions. However, some relevant research works, concerning acoustic signals emitted by real-life PD sources and recorded under on-site conditions should also be mentioned here; e.g., [32,33,34]. From the practical point of view, certain insufficiency may be observed regarding the investigations on the other AE sources (and signals that they emit) that may occur during acoustic PD measurements on working power transformers; minor papers deal with that issue. Even the latest guide of the Institute of Electrical and Electronics Engineers (IEEE) on the acoustic PD measurements does not discuss any of the potentially expected disturbances during on-site measurements [20]. On one hand, it, therefore, seems important to ensure that the recorded signals are actually emitted by the PD and not by any other source. On the other hand, if the captured signal is not generated by a PD, knowing the real source of it could be very helpful from the diagnostic point of view, especially when it is emitted by another (not PD) defect, occurred inside of the transformer. Therefore, the aim of this paper is to propose a two-step classification method for the identification of not only the selected PD sources but also other typical defects which may occur within a power transformer, and are accompanied by AE phenomena. The proposed method was evaluated on the basis of a database, containing AE signals, registered on real-life transformers, collected across several years and supplemented by some patterns of AE signals emitted by artificial PD sources. The applied signals were divided into eight classes corresponding to real AE signal sources diagnosed in working transformer units.

## 2. Research Methodology

**Figure 1:**
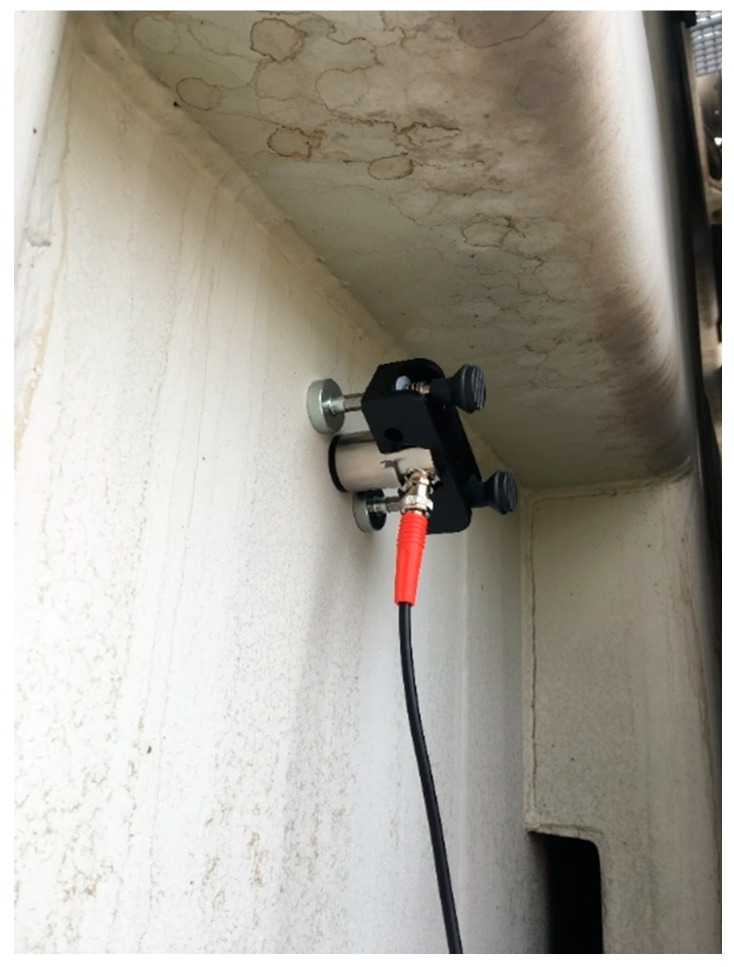
General view of the acoustic emission (AE) sensor during measurements on a 150 MVA power transformer.

The presented research was divided into three main stages: (1) signal registration, (2) initial analysis of the gathered data and (3) learning and verification of the selected classification algorithms. In the first stage of research works, acoustic signals were recorded using on-site working power transformers over several years. All of the signals used for this research were recorded by members of the same testing team, using the same metrological rigor and certified equipment described below. Moreover, only confirmed and confident signals were used in the research. Thus, the highest level of reliability has been supported. Typical AE measuring systems, commonly used for PD diagnostics, were applied for measurements, which include: a piezoelectric joint sensor, preamplifier, amplifier, and measuring interface. Different sensors were applied for registration, characterized usually by similar parameters; e.g., D9241A and R15α from PAC, AES75 from Omicron and VS75 from Vallen. In the case of sensors not equipped with integrated preamplifiers, an additional 2/4/6 preamplifier from PAC was used. In all scenarios, the AE2A from PAC amplifier and PicoScope type 5443B digital oscilloscope were used for signal registration. The typical frequency band for AE signals generated by PD under on-site conditions is expected to be in the range from 30 to 150 kHz. However, some researchers gave examples of PD signals where higher frequency components (300–400 kHz) were significantly presented [15,35,36]. Therefore, according to the current knowledge and authors’ experience, the upper frequency was set to 500 kHz. Thus, considering the Nyquist theorem, the sampling frequency applied for the measurements was set to 1 MHz. The signals were measured on the outer surface of the transformers tanks using a dedicated magnetic grip, for supporting constant and relevant clamp force and repeatable sensor and tank coupling (Figure 1). All of the on-site signals originated from units with a rating powers of 25–150 MVA, and voltages of 115/15 kV or similar. As mentioned above, apart from the real-life signals, also several PD defects, were simulated under laboratory conditions, in order to complement the existing database, created on the basis of field measurements. Finally, eight classes of typical sources of AE signals, corresponding to possible defects (or disturbance signals) expected in power transformers were proposed and investigated; assignments to particular classes are summarized in Table 1. Three classes are based on signals that were generated by artificial (lab) sources,

- PD generated in needle–needle electrode configuration in oil;
- PD generated in needle–plate electrode configuration in oil (grounded plate electrode);
- Surface PD in oil in rod–plate electrode configuration with pressboard paper between electrodes (grounded plate electrode).

Five further classes are based on signals emitted by real-life AE sources in on-site working power transformers, including the following.

- AE signals emitted by the core. Apart from typical low-frequency acoustic signals emitted by the transformer core, which are related to its vibrations (usually up to a few kHz) [6], ultrasound signals may be emitted as well. They are often related to magnetostriction phenomenon and/or loose elements around the iron sheets of the core; these kinds of defect have their dominant frequency bands at around 15–40 kHz [37].
- AEs emitted by oil flow. Such signals usually occur when the volume of the environment, in which the oil circulates, changes rapidly; e.g., around the joint of heat sinks with the main tank. A high turbulence occurs in such situations and depending on the density of the oil flow AE can be emitted. Such signals are usually expected in the vicinity of cooling system connections to the main tank or when some valves or locks do not function properly; e.g., they are not fully open or interfere with normal oil flow in any other way.
- AEs emitted by pumps. Oil pumps can typically be found in larger—high to medium voltage units, usually 40 MVA and above, where the oil-forced air-forced cooling system (OFAF) is the most common, unlike the oil-natural cooling system (ONAF) commonly used in smaller units, where pumps are not used. As pumps are rotating machines some typical AE signals are expected to be generated by them, especially by their worn parts: impeller, bearings, etc.
- AEs emitted by fans. These kind of signals are emitted by similar mechanisms as it is in case of pumps (fans are also rotating machines), but with the contribution of air (contrary to oil, in case of pumps). AEs emitted by a fan travels mainly in the steel (transformer tank) and may be easily received by an AE joint sensor. Such signals are treated as disturbances rather than useful signals; thus, they should be identified and distinguished from the other sources. However, some abnormalities in fans operation may also be revealed by them,
- AE emitted by PD. Exemplary signals emitted by internal PD in transformers during field tests were used as reference data in this class. Data from several different units representing surface PD (confirmed by other diagnostic methods) in oil were used.

Presence of all of the above-pointed defects sources was always confirmed by other advanced diagnostic methods; i.e., by means of electrical measurements, UHF measurements, dissolved gas method and/or internal inspection, etc. Therefore, the signals applied in this research are fully reliable.

In the second stage of research works, analyses including initial filtration (unification of sensors characteristics) and signal transformation using discrete wavelet transform (DWT) were performed, based on which energy patterns were assigned and the share of the total energy of the signal regarding each detail were calculated. In order to ensure proper signal interpretation, a relevant compensation of the characteristics of each sensor needed to be applied prior to further signal analysis. As characteristics of all of the sensors used in the research were known (provided by manufacturers), dedicated initial computations, based on an FIR (finite impulse response) filtration were applied according to the signals registered by each sensor respectively. As a result, further signal processing was performed on unified signals. Afterward, six levels of decomposition were proceeded, while the Symlets wavelet family was used. This type of wavelet is admitted to be one of the optimal solutions in the case of PD signal analysis. Although, sometimes Daubechies wavelets are also applied [15,27,38]. For further transformations, the wavelet filter order was set to 16, as it proved to be the optimal solution (higher filter orders did not bring any noticeable enhancements in minimizing the aliasing). The in the second stage, the research resulted in a set of six detail coefficients (cD1, …, cD6) for each of the signal samples, which was then applied in the next stage, for learning and classification purposes.

**Figure 2:**
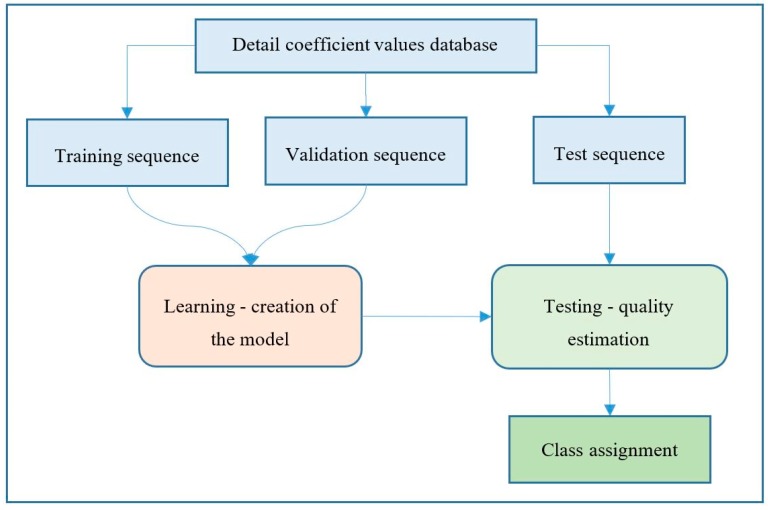
Block diagram of the learning and classification process.

The block diagram of general learning and classification process is presented in Figure 2. First, the input data we gathered (detail coefficient values, calculated for all signals in the database) was divided into three groups: training, validation and test sequences. Next, the classification model was created and then validated using the first two sequences. Finally, the model was tested using the third dataset, while the model quality was estimated. The process of learning and testing was repeated number of times, by adjusting model parameters, until optimal model quality was achieved.

**Figure 3:**
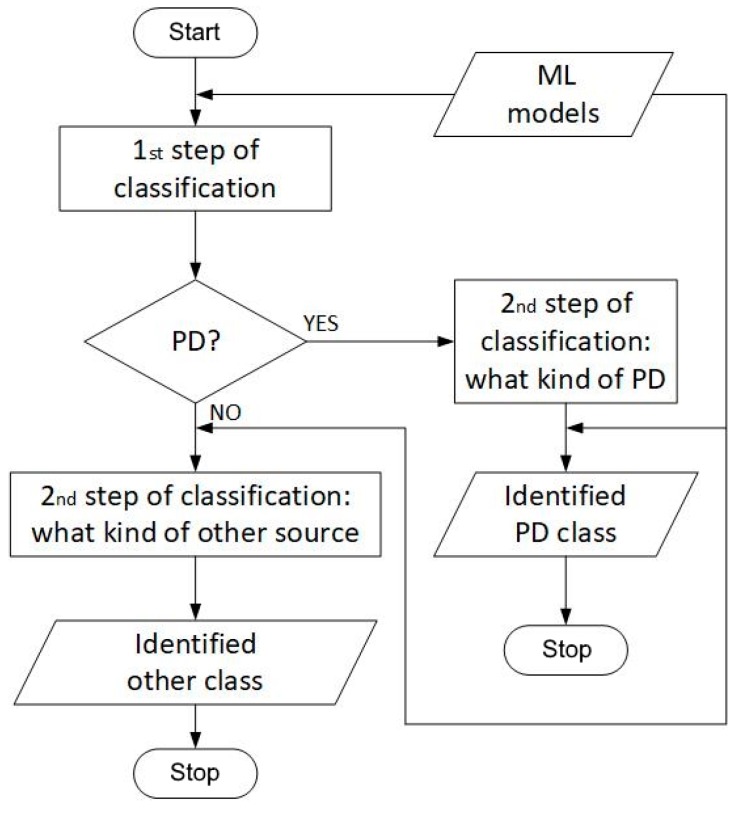
Flowchart of the method proposed for defect classification.

A two-step classification method was proposed in this paper. In the 1st step, it is indicated whether PD is present in the analyzed AE signal. In the 2nd step, it is determined what kind of PD or what other defect was identified based on the AE signal. The flowchart of the method is presented in Figure 3. Such a two-step classification method is currently expected for its potential application in a technical condition assessment system, because the crucial question is whether a PD occurs or not, and this may be answered already in the 1st step of the method.

As mentioned, in the two-step classification, a set of detail coefficients, calculated based on AE signals, corresponding to various source signals, was applied. In Table 2 the quantity of input data subjected to the process which were at our disposal, and the class symbol, are given. The large number of PD type samples results in its majority origin from laboratory measurements; e.g., 3514 AE signals were registered in the laboratory using a rod-plate electrode system with pressboard paper between electrodes (surface discharge—class C8). Obtaining measurements from real units is much more difficult; however, despite this fact, as will be shown in this paper, satisfactory results have been obtained in classifying defects even with a small number of samples (e.g., 20 samples for class C5).

For the purpose of the classification process, four different commonly known algorithms were applied:

- Binary decision tree for multiclass classification—fine tree model, marked as FT;
- Support vector machines using multiclass error-correcting output codes model with third-order polynomial kernel function—cubic SVM, marked as SVM;
- K-nearest neighbor classifier using Euclidean distance—fine KNN, marked as KNN;
- The ensemble of bagged decision trees—ensemble bagged tree, marked as EBT.

The process was performed using MATLAB and its Statistics and Machine Learning Toolbox. The options of the SVM classifier that were applied are as follows: The coding design defines for each binary learner, one positive and one negative class, and the rest is ignored by the software; this design exhausts all combinations of class pair assignments; the number of binary learners equals to k(k − 1)/2, were k is the number of classes. The kernel function is a polynomial of third-order. The predictors were standardized using their corresponding weighted means and weighted standard deviations.

Regarding the FT classifier applied options were as follows: The Gini’s diversity index was applied as a split criterion. The no of the maximal number of decision splits (branch nodes) was set as 100.

The options of KNN classifier we applied were as follows: The Euclidian distance without its weighting was applied as the distance metric; standardizations were applied; thus, the software centered and scaled each column of the predictor data by the column mean and standard deviation, respectively. Single nearest neighbor in dataset was used for classifying each point when predicting.

The options of EBT classifier chosen were as follows: For bagging, the bootstrap aggregation (bagging) and random forest algorithm were applied. The number of ensemble learning cycles was set to 30. The maximum number of decision splits for the bagged decision trees was set to 1423.

Cross-validation was included for all classifiers, in which the validation set was randomly partitioned into *k* = 20 sets. For each set, the algorithm reserved the set as validation data and trained the model using the other *k* − 1 set.

The research hypothesis was to confirm the possibility of using chosen classification methods to differentiate between processed AE signals from each other. The results are presented in the next section.

## 3. Results and Discussion

**Figure 4:**
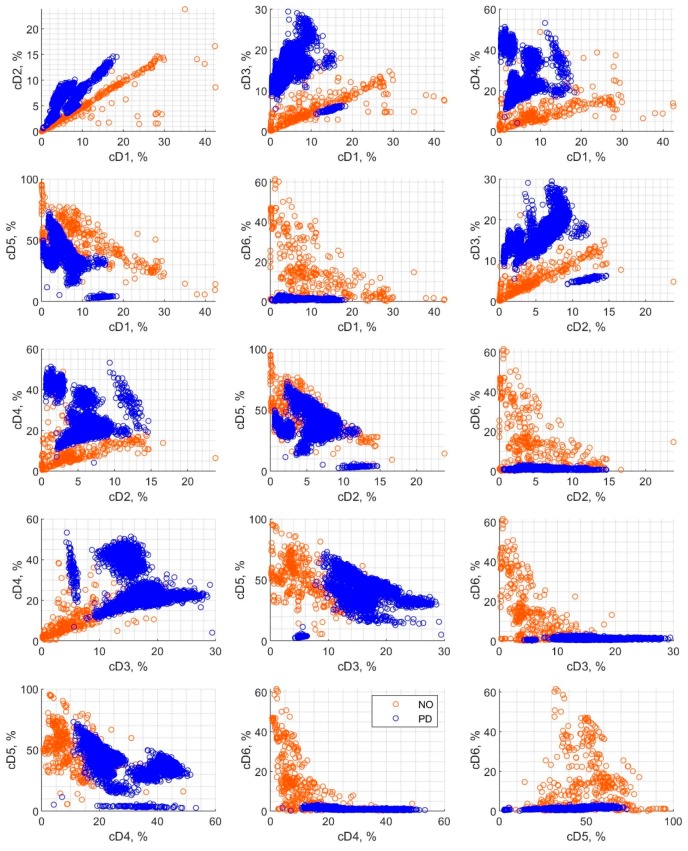
Scatter plot for detail coefficients cD1–cD6 depicted in pairs. The detail coefficients were applied for classification in the 1st step classification, with two classes: PD (partial discharge) and NO.

Figure 4 shows scatter plots of detail coefficients pairs, which were calculated using wavelet decomposition and divided into two classes for the 1st step of classification: “NO” (other source) and “PD”, (PD source) respectively. The colors correspond to the class applied for the classification process. The charts depict an exemplary representation of dependencies between the considered features. The points for the two classes in Figure 4 can be in many cases easily distinguished visually since there is practically no overlap between them. However, the division of points into two classes is not always trivial; hence different algorithms of machine learning are proposed in this paper to investigate their applicability to this task.

**Figure 5:**
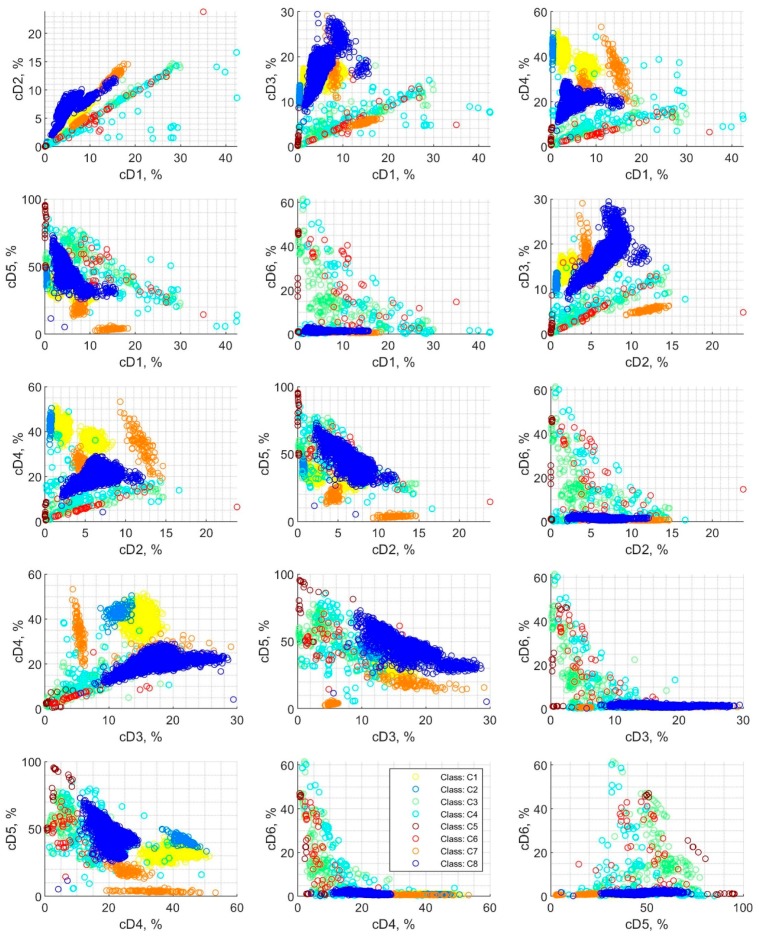
Scatter plot for detail coefficients cD1–cD6 depicted in pairs. The detail coefficients were applied for classification for 2nd step classification with eight classes: C1–C8.

Figure 5 shows scatter plots of detail coefficients pairs, which were calculated using wavelet decomposition and divided into eight classes for the 2nd step of classification. Additionally, in these charts, the colors correspond to the class applied for the classification process. These are exemplary representations of dependencies between the six features considered for classification. In contrast to the two-class case, the points for the eight classes in Figure 5 cannot be easily distinguished visually since there are many which overlap each other. The distribution of features is not always a coherent group, as is the case with class C1 and C8. For example, the dependencies for detail coefficients cD6 on the ordinate axis are strongly mixed and scattered over a wide area. Moreover, in some cases, the features are located in two groups of signals, which are separate in space, as is the case of class C7. This can be seen in many detail’s dependencies; e.g., cD3(cD1), cD4(cD1), cD5(cD4), cD5(cD3), cD4(cD3), cD5(cD1) and cD3(cD2).

**Figure 6:**
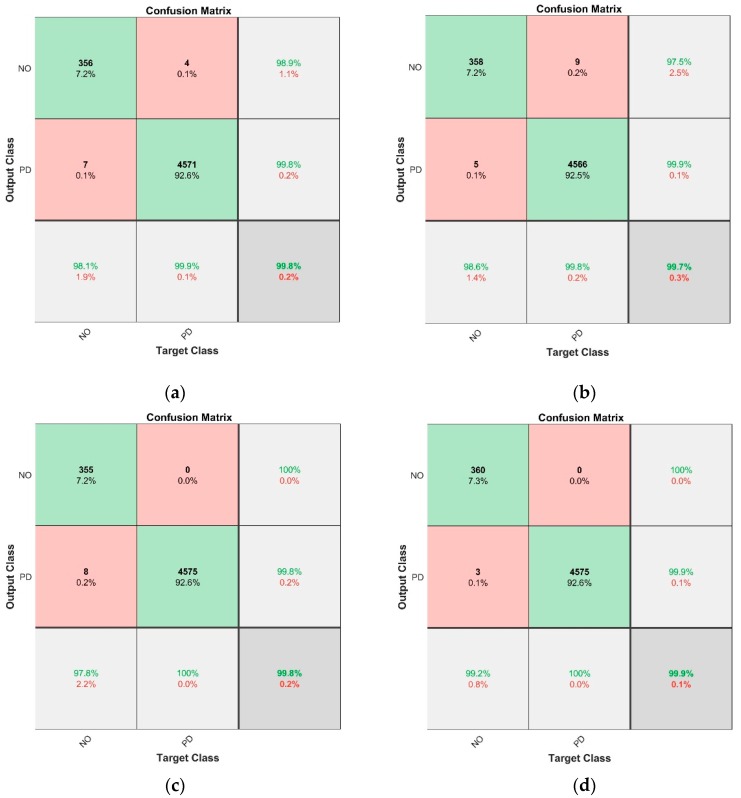
Confusion matrices calculated based on 1st step classification results: (**a**) EBT model; (**b**) FT model; (**c**) KNN model; (**d**) SVM model.

**Figure 7:**
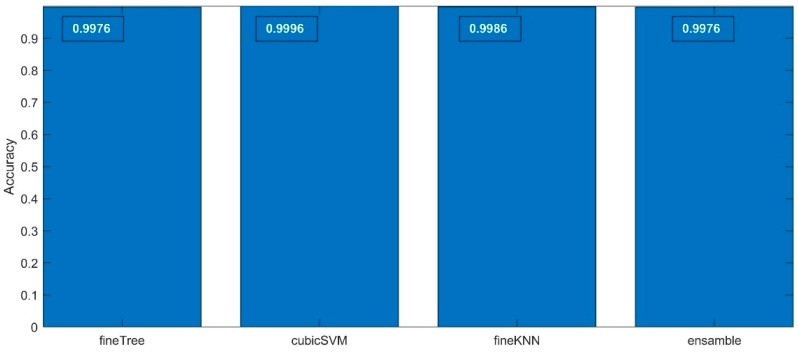
The cross-validated accuracy values calculated for the four classification methods applied for the 1st step classification process.

The six calculated detail coefficients: cD1–cD6, were submitted for classification purposes. In the 1st step, when dividing the signals into two classes, corresponding to AE signal emitted by PD or other sources, very good results were achieved, which was confirmed by values depicted in the confusion matrix (Figure 6) and by the accuracy values (Figure 7).

Figure 6 depicts the metrics calculated for the four classification methods: (a) EBT model; (b) FT model; (c) KNN model; (d) SVM model. The bold values indicate the number of cases the algorithm calculated the given class. The values given right below correspond to the overall percentages. The last row of the chart includes green and red values which are the true positive rates (TPR) and the false-positive rates (FPR), respectively. The last column includes in green the positive predictive rates (PPR) and in red the false discovery rates (FDR). The cell in the bottom right corner of the matrix depicts the overall accuracy (in green) and error rate (in red). Based on the metrics the following can also be stated,

- For the EBT model (Figure 6a)—the “NO” class was incorrectly classified as “PD” class in only seven of 363 cases, while the “PD” class was wrongly classified as “NO” class in only four of 4575 cases;
- For the FT model (Figure 6b)—the “NO” class was misclassified as “PD” class in only five of 363 cases, while the “PD” class was misclassified as “NO” class in only nine of 4575 cases,
- For the KNN model (Figure 6c)—the “NO” class was incorrectly classified as “PD” class in only eight of 363 cases, while the “PD” class was never misclassified,
- For the SVM model (Figure 6d)—the “NO” class was incorrectly classified as “PD” class in only three of 363 cases, while the “PD” class was never misclassified.

It may be clearly seen that for all algorithms the matching values exceeded 99.7%. Nevertheless, it can also be stated that the SVM model (Figure 6d) had the best values.

The signals corresponding to the PD phenomena are overrepresented compared to the “NO” class, which corresponds to other phenomena emitting AE signals. This issue does not have a great impact on the calculation results though, since for the prediction process stratified 20-fold cross-validation was applied, which assures that all test sets include observation from all classes.

The accuracy values of the classification results are presented in Figure 7—the exact accuracy values are marked in the figures upper section in white. All the algorithms used have a very high match, having values exceeded 99%. The differences between them are insignificant. For the details of the analysis, it can be said that the best fit was obtained by the SVM algorithm, although it may be states that each of the algorithms is able to solve the specified classification task.

**Figure 8:**
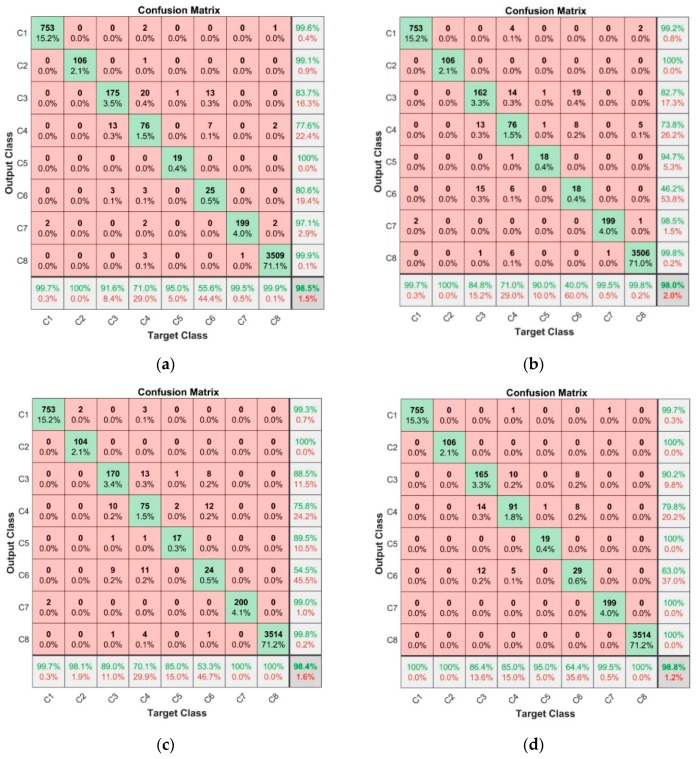
Confusion matrices calculated based on 2nd step classification results using: (**a**) EBT model; (**b**) FT model; **(c**) KNN model; (**d**) SVM model.

Figure 8 depicts the calculated confusion matrices based on 2nd step classification results for the four classification methods applied: EBT model (Figure 8a); FT model (Figure 8b); KNN model (Figure 8c); SVM model (Figure 8d), respectively. As may be explicitly observed, in all cases the matching (accuracy) values exceeded 98%. In general, all four models had similar results with an error rate not exceeding 2.0%, but, after splitting the classifiers into eight classes, noticeable differences in classification goodness for particular classes were observed. For example, the arithmetic mean value over TPR and PPR *µ,* exceeded 98.8% for classes: C1, C2, C7 and C8; *µ* = 93.5% for class C5; and *µ* = 86.6% for class C3, which indicates optimal classification results. The number of samples is not critical in this process, as it may be seen based on comparison between class C5 (where only 20 samples were available) and class C3 (where, having nine times more data, inferior classification results were achieved). The worst quality metrics were gathered for classes C4 and C6 with *µ* = 75.1% and *µ* = 56.8%, respectively.

**Figure 9:**
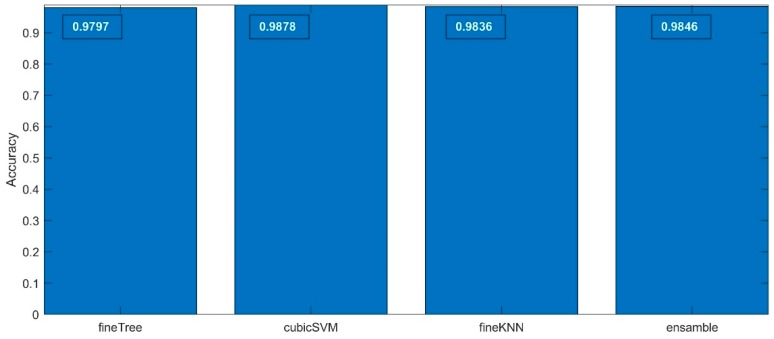
The cross-validated accuracy values calculated for the four classification methods for the 2nd step classification process.

The accuracy values of the results gathered in 2nd step classification process are presented in Figure 9, where the exact values are depicted in the figures upper section in white. In all cases, the accuracy value exceeded 0.97, proving very good classification results. Although the differences are very similar and the values differ in the hundredth part, we can state that also, in this case, the SVM model (cubicSVM) reached the best rating.

## 4. Conclusions

In this paper, a new approach for ML-based classification of selected defects occurring in power transformers is presented. The proposed method utilizes AE measurements, and according to the results presented in this paper, it significantly extends practical applications of this kind of PD detection in terms of power transformers’ technical condition assessments. The classification process is divided into two steps: the 1st step provides information on whether the analyzed signal is emitted by PD or by another AE source; the 2nd step enables the identification of the particular AE source type. As the proposed method is based on AE signals registered on real-life working transformers, which represent its typically diagnosed defects or disturbances, the results achieved are reliable. The most essential contributions of research presented in the paper are as follows.

- The classification method allows identifying three types of PD occurring in oil as well as four further defects that may occur within the diagnosed power transformer;
- High precision of the classification method has been confirmed by means of accuracy measurement, which exceeded 0.98, regarding all of the ML algorithms applied;
- All of the applied ML models yielded similar results with error rates not exceeding 2.0%, but, after splitting the classifiers into eight classes, noticeable differences in classification goodness for particular classes were observed. The reasons for these slight differences in classification effects lie in the differences in the operation of each algorithm;
- The best goodness of classifications, expressed by mean values over TPR and PPR, were from classes C1, C2 and C7, while the worst was from class C6;
- The size of training sequences may have an impact on the classification results, but does not determine them fully, as was observed for class C5, where underrepresentation of the training sequence did not influence the results significantly;
- Differences in classification results were observed for classes C2 and C4 when different classification results were obtained for similar database sizes;
- The overall highest goodness measure was achieved for the SVM algorithm;
- DWT based energy patterns applied as input signals have proven to be effective predictors for defect classification. Determining which detail is more or less important is a separate issue. This type of analysis is not in the content of this article, because the task of classification did not require high computational power and it was not necessary to reduce the number of features,
- The proposed two-step classification method is currently expected for its potential application in a technical condition assessment system, as the crucial question is whether a PD occurs or not, and this may be answered already in the 1st step of the proposed method;
- The method may be relatively easily extended for identification of other defects in the future. The required steps include the delivery of the learning sequence and execution of the learning process according to the procedure shown in Figure 2.

## Figures and Tables

**Table 1:** Summary of the signals source type and assignment to classes.

Class Symbol	Corresponding AE Source	Class Group	Signal Origin
C1	needle-needle	PD	Artificial (lab)
C2	needle-plate	PD	Artificial (lab)
C3	Core	Other	Real-life (Transformer)
C4	Oil flow	Other	Real-life (Transformer)
C5	Fans	Other	Real-life (Transformer)
C6	Pumps	Other	Real-life (Transformer)
C7	Surface PD	PD	Real-life (Transformer)
C8	Surface PD	PD	Artificial (lab)

**Table 2:** Number of samples applied for the learning/validation/testing process.

Classification Step No	Class Symbol	No of Samples Applied
1st	PD	4575
1st	NO (other)	363
2nd	C1	755
2nd	C2	106
2nd	C3	191
2nd	C4	107
2nd	C5	20
2nd	C6	45
2nd	C7	200
2nd	C8	3514
